# Multiobjective Particle Swarm Optimization Based on Cosine Distance Mechanism and Game Strategy

**DOI:** 10.1155/2021/6440338

**Published:** 2021-11-06

**Authors:** Nana Li, Yanmin Liu, Qijun Shi, Shihua Wang, Kangge Zou

**Affiliations:** ^1^School of Data Science and Information Engineering, Guizhou Minzu University, Guiyang 550025, China; ^2^School of Mathematics, Zunyi Normal University, Zunyi 563002, China; ^3^School of Mathematics and Statistics, Guizhou University, Guiyang 550025, China

## Abstract

The optimization problems are taking place at all times in actual lives. They are divided into single objective problems and multiobjective problems. Single objective optimization has only one objective function, while multiobjective optimization has multiple objective functions that generate the Pareto set. Therefore, to solve multiobjective problems is a challenging task. A multiobjective particle swarm optimization, which combined cosine distance measurement mechanism and novel game strategy, has been proposed in this article. The cosine distance measurement mechanism was adopted to update Pareto optimal set in the external archive. At the same time, the candidate set was established so that Pareto optimal set deleted from the external archive could be effectively replaced, which helped to maintain the size of the external archive and improved the convergence and diversity of the swarm. In order to strengthen the selection pressure of leader, this article combined with the game update mechanism, and a global leader selection strategy that integrates the game strategy including the cosine distance mechanism was proposed. In addition, mutation was used to maintain the diversity of the swarm and prevent the swarm from prematurely converging to the true Pareto front. The performance of the proposed competitive multiobjective particle swarm optimizer was verified by benchmark comparisons with several state-of-the-art multiobjective optimizer, including seven multiobjective particle swarm optimization algorithms and seven multiobjective evolutionary algorithms. Experimental results demonstrate the promising performance of the proposed algorithm in terms of optimization quality.

## 1. Introduction

In the field of engineering, aviation scheduling, optimal control, and others, most of the optimization problems are multiobjective optimization problems (MOPs) [1]. MOPs are different from single objective optimization problems. More objective functions need to be optimized, which have the characteristics of conflict or influence each other [2]. This means that it is impossible for all the objective function values to be optimal, in which the optimal solution for one objective function may be the worst solution for another objective. Therefore, a set of trade-off solutions, known as Pareto optimal set, is adopted to represent the best possible compromises among objectives in MOPs. The practical problems are considered to have the characteristics of high-dimensional nonlinearity and strong constraints, so classic optimization algorithms (conjugate gradient method [3], Newton method [4], simplex algorithm [5], etc.) can no longer solve MOPs effectively. With the development of science and technology, the emergence of intelligent control makes multiobjective optimization reach a more advanced stage. The method of optimal control conditions can take different paths. For example, the introduction of the deformable MEMS device in [6] has a positive effect on improving optimal control. In addition, the intelligent optimization algorithm, which belongs to the bionic algorithm, has also attracted the attention of researchers. Among them, the particle swarm optimization (PSO) algorithm [7], which has the advantages of simple operation, fast velocity, wide application range, and few setting parameters, has become the focus of more researchers.

PSO derived from the simulation of complex adaptive systems which was an evolutionary computation method based on swarm intelligence was proposed by Kennedy and Eberhart in 1995. It was developed inspired by the social behavior of a swarm of animals like birds. In PSO, individuals were called particles and each particle represents a potential solution. The swarm consists of a group of particles flying through the search space searching for the optimal solution, like birds searching for food. Individuals called “particles” in PSO “flow” through the ultradimensional search space. The position change of particles in the search space was based on the individual's social and psychological intention surpassing other individuals successfully. It could communicate with other individuals and change its structure and behavior according to the process of “learning” or “accumulating experience.” Therefore, changes in the velocity and position of particles will be affected by the experience of other particles.

With the development of intelligent algorithms, the relative simplicity and the practical success of single objective optimizer have motivated researchers to extend the usages of PSO from the single objective optimization problems into MOPs. In 2002, Coello et al. extended PSO from a single objective to multiple objectives, which was used to solve MOPs for the first time [8]. In the research of multiobjective particle swarm optimization (MOPSOs), there are at least two fundamental issues to be addressed. The first issue is how to use a standard to select excellent global leader as the learning sample of all particles flight to guide other particles in the population. Due to the important influence of the leader in the search direction, the random selection of global learning samples in the external archives may lead the algorithm to be trapped into a local optimum. At present, most of MOPSOs based on dominance use the infinite external archive to store nondominated solutions, so the maintenance and update of the external archive are also very important. The second issue is how to balance convergence and diversity of the swarm. It is crucial to the performance of MOPSOs, because PSO-based multiobjective optimizations are very likely to be trapped into the local optimum (or one of many optima) of MOPs due to their fast convergence.

In this article, a novel multiobjective particle swarm optimization based on cosine distance mechanism and game strategy was proposed, which was called GCDMOPSO. To maintain the update mechanism of the external archive, the cosine distance was used to delete the worst particles in the external archive. At the same time, the same number of particles was selected in the candidate set to supplement the deleted particles in the external archive to maintain the update of the external archive dynamically. The main contributions of this article were as follows:

1. Dynamic maintenance of the external archive updates. After each iteration of the algorithm, the nondominated solutions selected from the candidate set were added to the external archive. When the number of nondominated solutions in the external archive exceeded the maximum size, the cosine distance was used to compare the degree of crowding of the nondominated solutions in the archive, and the most crowded solutions were deleted. Then, it could also identify the removed solutions and update the crowding degree of all solutions in the domain (i.e., after deleting the most crowded solutions, recalculate the cosine distance of all other solutions). This method achieves better diversity and preservation.
2. The method by which the individual was selected. In the update process of this algorithm, the fitness value of each individual was calculated through nondominated sorting, which will generate individuals of the same ranking value. The individuals with the same ranking value were selected into the candidate set, and the Euclidean distance between each individual and the origin of the coordinate was calculated. Then the Euclidean distance from each individual to the coordinate origin was sorted in ascending order. In order to maintain the updating of external archive dynamically, when we delete the particles in the external archive, we need to put the same number of individuals into the archive.
3. The selection of the global leader. Based on the recently developed competitive group optimizer and combined game mechanism, this article proposed a novel global leader selection strategy based on the game mechanism. Randomly select two nondominated solutions in the external archive, and compare the cosine distances of the two nondominated solutions, respectively. The winner was selected as the global leader, leading other particles to fly. We can keep all obtained solutions converging along the real Pareto front.

The remaining part of this article is structured as follows.  Section 2 describes the related definitions of the MOPs and MOPSOs briefly, as well as the related works from which the main ideas are inspired for designing the new algorithm in this article. Then, the details of the proposed GCDMOPSO are described in Section 3.  Section 4 is the experimental part of GCDMOPSO. GCDMOPSO is compared with some selected MOPSOs and MOEAs in this article. Finally, the conclusions are drawn in Section 5.

## 2. Background

### 2.1. MOPs

In this section, the definition of the fundamentals of the MOPs is presented. The mathematical forms of the MOPs are described as follows:

(1) $$\begin{array}{c} \min y=F\left(x\right)=\left(f_{1}\left(x\right),f_{2}\left(x\right),\ldots ,f_{m}\left(x\right)\right)\\ \text{s.t.}\;\left\{\begin{array}{c} g_{i}\left(x\right)\geq 0,\;i=1,2,\ldots ,p\\ h_{j}\left(x\right)=0,\;j=1,2,\ldots ,q, \end{array}\right. \end{array}$$

where *x*=[*x*_1_, *x*_2_,…, *x*_*d*_,…, *x*_*d*_] is the decision vector of *D* dimension, *x* ∈ *X*, and *X* is the decision space; *x*_*d*_min_ ≤ *x*_*d*_ ≤ *x*_*d*_max_, *d* = 1, 2,…, *D*; *x*_*d*_max_ and *x*_*d*_min_ are the upper and lower bounds of each dimension vector; *y* is the objective vector, *y* ∈ *Y*, and *Y* is the objective space; *m* is the total number of optimization objectives; {*g*_*i*_(*x*) ≤ 0} is the *i* − th inequality constraint; *h*_*j*_(*x*)=0 is the *i* − th equality constraint. These two constraints determine the feasible region of the solution.

MOPs are different from single objective problems, so the same problem-solving ideas cannot be adopted by the former. It is impossible for a certain solution of MOPs to achieve optimal results for all objectives at the same time, and different solutions cannot be compared due to different objective functions. Therefore, when solving a MOP, a set of solutions are usually obtained, and these solutions have different effects for different objective functions. The solutions in this set are called the nondominated solutions or Pareto optimal solutions. The following is a detailed introduction to the related concepts.

**Definition 1 .**

Pareto dominance, *x*_*u*_ ∈ *X*, *x*_*v*_ ∈ *X*, *X* are two feasible solutions of this MOP, and *x*_*v*_ is dominant by comparison with *x*_*u*_, expressed as *x*_*v*_≺*x*_*u*_, if and only if

(2) $$\begin{array}{c} \forall k\in \left\{1,2,\ldots ,m\right\},f_{k}\left(x_{v}\right)\leq f_{k}\left(x_{u}\right)\wedge \exists l\in \left\{1,2,\ldots ,m\right\},f_{l}\left(x_{v}\right)<f_{l}\left(x_{u}\right). \end{array}$$

**Definition 2 .**

For Pareto optimal, *x*^*∗*^ ∈ *X* is the Pareto optimal solution on *X*, if and only if the following conditions are satisfied

(3) $$\begin{array}{c} \neg \exists x\in X:x≺x^{*}. \end{array}$$

That is, there is no better solution than *x*^*∗*^ in the set *X*, so *x*^*∗*^ is the optimal solution in *X*, which is also called nondominated solution or noninferior solution.

**Definition 3 .**

For Pareto optimal set, for the MOPs, the optimal solution set can be defined as follows:

(4) $$\begin{array}{c} p^{*}=\left\{x\in X|\exists x^{\prime }\in X,f_{k}\left(x\right),\left(k=1,2,\ldots ,m\right)\right\}. \end{array}$$

**Definition 4 .**

For Pareto optimal front, the curved surface consisting of the objective function values corresponding to all Pareto optimal solutions in Pareto optimal solution set is called Pareto front:

(5) $$\begin{array}{c} \text{PF}=\left\{F\left(x^{*}\right)=\left(f_{1}\left(x^{*}\right),f_{2}\left(x^{*}\right),\ldots ,f_{m}\left(x^{*}\right)\right)|x^{*}\in p^{*}\right\}. \end{array}$$

### 2.2. Multiobjective Particle Swarm Optimization

MOPSO is an improvement of PSO. In PSO, the individual birds in the population are abstracted as massless particles. Each particle has its own velocity and position. The position and velocity of *i* particle are expressed as *x*_*i*_=(*x*_*i*1_, *x*_*i*2_,…, *x*_*i*  *D*_) and *v*_*i*_=(*v*_*i*1_, *v*_*i*2_,…, *v*_*i*  *D*_), respectively. Searching for food in *N* is the space, and food is considered as the optimal solution. Particles are updated according to the following formula:

(6) $$\begin{array}{c} v_{i}\left(t+1\right)=wv_{i}\left(t\right)+c_{1}r_{1}\left({\text{pbest}}_{i}\left(t\right)-x_{i}\left(t\right)\right)+c_{2}r_{2}\left({\text{gbest}}_{i}\left(t\right)-x_{i}\left(t\right)\right), \end{array}$$

(7) $$\begin{array}{c} x_{i}\left(t+1\right)=x_{i}\left(t\right)+v_{i}\left(t+1\right). \end{array}$$

The right side of equation (6) consists of three parts. The first part is the inertia quantity, where *w* is the inertia weight. Its size determines how much the particle inherits to the current velocity. If the value of *w* is large, the overall search capability of the algorithm will be enhanced; if the value of *w* is small, the local search function of the algorithm will be improved. *w* is generally limited to a random number less than 1. The second part is the cognition of the individual, which represents the movement of the individual to the best position according to his historical flight experience. Among them, pbest represents he optimal position of the individual, *r*_1_ is a random number normally distributed in the interval (0, 1), and *c*_1_ is the learning factor, representing the degree of particles learning. The third part is the amount of social cognition, which leads to the amount of particles that move to the global optimal position. gbest represents the global optimal position, *r*_2_ is a random number normally distributed in the interval (0, 1), and *c*_2_ is the learning factor, where *c*_1_=*c*_2_=2 is usually taken. The coordination of these three parts determines the overall performance of the algorithm.

With the deepening of research, many scholars have extended PSO to MOPSO, so that the algorithm is more suitable to solve MOPs. In MOPs, the number of the optimal solutions is not unique due to the increase of constrained objective. Combined with PSO, the difference of MOPSO is not only the selection of the historical optimal position and the global leader under multiple constraints but also the storage of the historical optimal position and the global leader. Therefore, MOPSOs used the external archive mechanism to solve storage problems and used the external archive to save the nondominated solutions generated during the search in the entire swarm. The nondominated solutions in the external archive are not dominated by any other particles in the external archive. Therefore, all nondominated solutions in the external archive should meet the two following requirements: (a) The nondominated solutions in the external archive collection do not have a mutual dominance relationship, and it is impossible to compare which of the nondominated solutions are better. (b) The introduced particles are stronger than the solutions in the original external archives, and the weaker solutions in the original external archives should be eliminated.

### 2.3. Existing MOPSOs

The first PSO variant was proposed by Coello et al. [9]. The authors incorporated the concept of Pareto advantage into the method of PSO. The local optima and the global optima in the swarm were determined by the Pareto dominance principle. For the first time, the secondary storage library (i.e., the external archive) was used to store the nondominated solutions obtained after each iteration. This was the first time that PSO has been used to solve MOPs. Compared with classic MOEAs such as NSGA-II [10] and PAES [11], the first MOPSO proposed was more competitive in solved MOPs, but it was unable to solve MOPs with complex landscapes. To address this issue, Sierra and Coello et al. [12] proposed an improved PSO-based multiobjective optimization, in which Pareto advantage and congestion factor were used to select a list of available leading solutions; and the swarm was divided into three subswarms simultaneously; then different mutation operators were suggested for different subswarms divided by users in advance. In addition, the experience of this algorithm used *ε* dominance to fix the size of the external archive. Experimental results show that the performance of the improved optimization on MOPs with multiple local fronts is more competitive.

A speed-constrained MOPSO was proposed by Nebro et al., called SMPSO [13], in which the velocity of all particles was restricted in order to tackle MOPs with multimodal landscapes. The SMPSO allowed new effective particle positions to be generated when the velocities were too large. Other features of the SMPSO included polynomial mutation as turbulence factor and the external archive was comprised of nondominated solutions which were found during the search process. However, most of MOPSOs could not solve MOPs effectively due to the fact that velocities in such algorithms were too rapid.

The above MOPSOs only used a single search strategy to update particle's velocity. So, Lin et al. proposed a novel MOPSO based on multiple search strategies [14], which used a decomposition method to transform MOPs into a set of aggregation issues, and then allocated each particle accordingly to optimize each aggregation issue. This algorithm designed two search strategies to update the velocity of each particle. After that, all nondominated solutions visited by particles were preserved in an external archive, and the evolutionary search strategy was further executed to exchange useful information between them. These multiple search strategies enabled this novel MOPSO to handle various MOPs more effectively.

In contrast to the MOPSOs where the global optimal solution is determined by dominance relations, Zhang and Li used the framework of MOEA/D [15] to try to embed the decomposition mechanism into the PSO-based multiobjective optimization for the first time and proposed a MOPSO by decomposing a MOP into a number of single objective optimization problems [16]. The algorithm used the PSO search method instead of the genetic operator. Later, an improved version of this multiobjective optimization called SDMOPSO [17] was proposed by Al Moubayed et al. In SDMOPSO, the global optima were only selected from the neighborhood of particles, and crowded files were used to preserve the diversity of swarm leaders. Dai et al. divided the solution space into multiple subspaces and retained only one optimal solution in each subspace so that the nondominated solutions can be evenly distributed. This MOPSO was based on object space decomposition [18]. Based on the decomposition method, Martłnez and Coello also proposed a version of multiobjective optimization called dMOPSO [19], in which the global leader was determined according to the scalar aggregation value. Moreover, a memory reinitializationstrategy was used when a particle reached a certain value. The main aim of this approach was to preserve diversity and to avoid trapping in local fronts. Although the improvement of this algorithm holds a lower computational cost than most of the other MOPSOs which often need to maintain an archive, it is difficult to converge to the true Pareto front when dealing with complex models.

In 2020, Alkebsi and Du proposed a novel MOPSO. This algorithm was a novel archive update mechanism based on the nearest neighbor method, called MOPSONN [20]. In the early stage of this algorithm, the external archive was updated based on the nearby distance measurement. In later generations, two new rules were used, namely, the maximum cost rule and the cost sum rule, to update the archive. These two archive update strategies updated the nondominated solutions in the archives.

In addition, a few scholars have improved the MOPSOs from the aspect of parameter setting to make the MOPSO more optimized [21]. In view of the effective analysis of the abovementioned existing algorithms, this article combined with the cosine distance update mechanism and the meshing strategy. A novel multiobjective game particle swarm optimization based on the cosine distance update mechanism was proposed, which effectively improves the convergence and diversity of solving MOPs. The following section describes the proposed algorithm in detail.

### 2.4. Acronyms in the GCDMOPSO

In order to read the article more clearly, a table of acronyms is listed in this article. The specific contents are shown in Table 1.

## 3. The Proposed the GCDMOPSO

In this section, the details of our proposed GCDMOPSO are introduced. The algorithm generates a new population from all individuals initialized randomly. The particles of this population will generate many levels according to their dominance relationship. The first-level individuals generated by the nondominated relationship flow into the candidate set, and a new external file is further created. Then, based on the grid technology and the cosine distance strategy, the individuals introduced in the candidate set are screened to dynamically maintain the external archive. At the same time, the nondominated solutions in the external archives are screened through game strategy as the global leader to guide other individuals to fly. After that, this program updates the velocity and position of the group according to equations (6) and (7).

### 3.1. Selection of Introduced Particles

**Figure 1:**
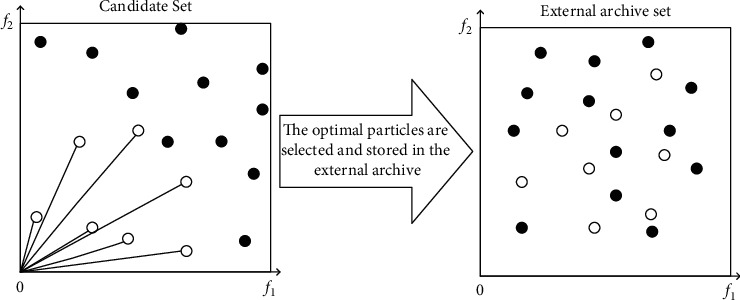
Schematic diagram of selecting introduced particles.

Any individual only chooses the appropriate type of talents as the learning object, and only the outstanding individuals will be selected into the external archives as leaders to lead other individuals to update and iterate. According to the previous MOPSOs, the program calculated the fitness value of each individual and randomly selected individuals with the same ranking value as candidate solutions to enter the external archive to guide other individuals to fly. Due to the fact that the fitness value was calculated to generate the first-level ranking value after the iterative update of the algorithm may have the same value, the random selection method in the previous algorithm could not better select the candidate solution. This article has improved it in this part. As shown in Figure 1, in our algorithm, a candidate set is added. The fitness value of each individual is calculated, and the first-level individuals flow into the candidate set. At the same time, the candidate set is regarded as a grid, and the Euclidean distance from the fitness value of each individual to the origin of the coordinate is recalculated. Then the distance from each individual to the origin of the coordinate is sorted in ascending order, and individuals closer to the origin of the coordinates are selected into the external archive. If the nondominated solutions in the external archive do not reach the maximum size, all individuals in the candidate set are entered into the external archive according to the individual fitness ranking value, and they are stored; if the nondominated solutions in the external archive reach the maximum size, the individuals with the smaller cosine distance in the external archive will be eliminated.

In other words, in order to maintain the number of particles in the external archive mechanism at stable level, when a certain number of particles are deleted, the same number of particles will be added from the candidate set.

### 3.2. Maintenance and Update of External Archives

Archiving strategy is an important part of MOPSOs. Excellent maintenance capabilities can not only improve the search efficiency of the algorithm but also improve the convergence of the algorithm on the other hand. This article mainly adopts the external archive scheme to store the nondominated solutions generated during the entire iterative update. The maintenance principle of the external archive mainly uses the cosine distance measurement mechanism. The cosine distance measurement mechanism is usually used in the field of text classification. Since the text space and the multiobjective space are both multidimensional spaces, they have certain similarities at the same time. Therefore, the cosine distance measurement mechanism is applied to the multiobjective optimization. If a dimension is represented by a vector, the dimension of a vector can be regarded as a single objective. The cosine distance between objectives can be used to determine the density relationship between individuals.

**Definition 5 .**

For weight ratio, suppose that the population size is *N* and the objective function value of particle *i* is expressed as *f*_*i*1_(*x*), *f*_*i*2_(*x*),…, *f*_*ik*_(*x*),…, *f*_*im*_(*x*). For the *i* − th particle, the weight ratio of the objective function value in the *k* dimension is as follows:

(8) $$\begin{array}{c} W_{ik}=\frac{f_{ik}\left(x\right)}{\sum_{i=0}^{N}f_{ik}\left(x\right)}. \end{array}$$

**Definition 6 .**

For cosine distance, suppose that the objective vector of any particle *i* is expressed as *d*_*i*_=(*W*_*i*1_, *W*_*i*2_,…, *W*_*ik*_,…, *W*_*im*_); according to the cosine formula, the cosine distance between two objectives is

(9) $$\begin{array}{c} \text{CD}\left(d_{i},d_{j}\right)=1-\cos\left(d_{i},d_{j}\right)\\ =1-\frac{\sum_{k=1}^{m}W_{ik}\times W_{jk}}{\sqrt{\sum_{k=1}^{m}{\left(W_{ik}\right)}^{2}}\times \sqrt{\sum_{k=1}^{m}{\left(W_{jk}\right)}^{2}}}. \end{array}$$

**Figure 2:**
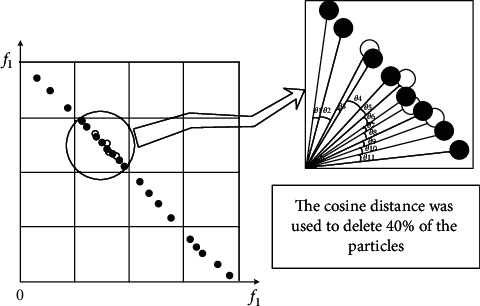
Example diagram of nondominated archive deletion outside.

In this article, in order to better control the size of the external archive, the size of the external archive is set to 200. As shown in Figure 2, the objective space is divided into *k* subregions. Then a subregion with highest density is selected, and the cosine distance between each nondominated solution in each subspace and its neighboring particles is compared. The smaller cosine distance between the nondominated solution and its neighboring particles, the greater the density of the nondominated solution and the poorer distribution.

The GCDMOPSO calculates the cosine distance between the nondominated solution and its neighbor particles according to Definitions 5 and 6 and sorts the cosine distance in ascending order. Then, the nondominated solutions with minimum cosine distance, minimum angle, and maximum density are selected for dynamic deletion. In addition, only one nondominated solution is deleted. Then the cosine distances of other nondominated solutions are recalculated, and the nondominated solution with the smallest cosine distance is deleted. The solid black dots are the remaining nondominated solutions, and the hollow circles are the deleted individuals, with a deletion rate of 40%. At the same time, the same number of individuals is selected in the set of candidate solutions to supply the nondominated solutions deleted in the external archive to maintain the update of the external archive.

**Figure 3:**
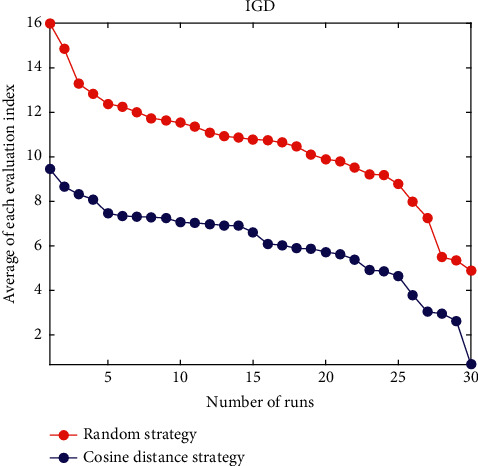
Schematic diagram of comparison between random strategy and cosine distance strategy.

Leaders guiding the optimization process are an effective way to design MOPSOs. Among the many strategies currently available, the direction that prompts particles to explore some potential areas guides the search. The cosine distance strategy proposed in this article is quite different from the random strategy proposed in the past.  Figure 3 shows a schematic diagram of the comparison between the cosine distance strategy and the random strategy. First of all, all the evaluation indicators of the two strategies (ZDT1–ZDT4 and ZDT6, DTLZ1–DTLZ5, UF1–UF10) are run 30 times, respectively. The data of all evaluation indicators running 30 times are sorted in descending order into 30 levels. Then all the evaluation indicators of each level are averaged. The ordinate indicates the average of all evaluation indicators for each level, and the abscissa indicates that each strategy has been run 30 times. It can be seen from Figure 3 that, in the same level, the average of the cosine distance strategy is better than the average of the random strategy significantly, which fully illustrates the feasibility of the cosine distance strategy.

**Figure 4:**
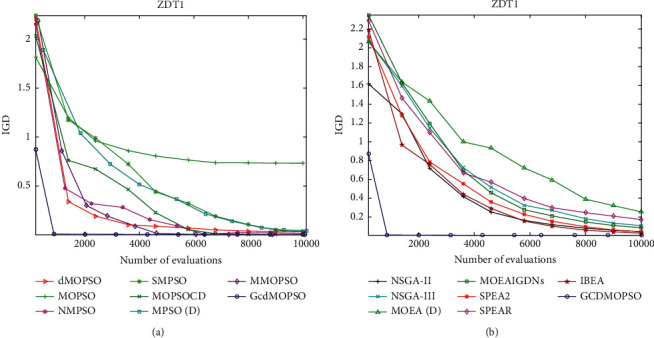
Illustration for detecting the evolutionary state by the cosine distance strategy. (a) The convergence trajectory of MOPSOs on ZDT1. (b) The convergence trajectory of MOEAs comparison on ZDT1.

Figure 4 shows that the GCDMOPSO used the cosine distance strategy to detect the evolution state. Taking ZDT1 as an example, it was compared to seven state-of-the-art MOPSOs and seven classic MOEAs on ZDT1. (a) shows the convergence trajectory of the GCDMOPSO and seven MOPSOs on ZDT1; (b) shows the convergence trajectory of the GCDMOPSO and seven MOEAs on ZDT1. The experimental results indicate the promising convergence speed of the proposed GCDMOPSO in comparison with the seven state-of-the-art MOPSOs and seven classic MOEAs on ZDT1.

**Figure 5:**
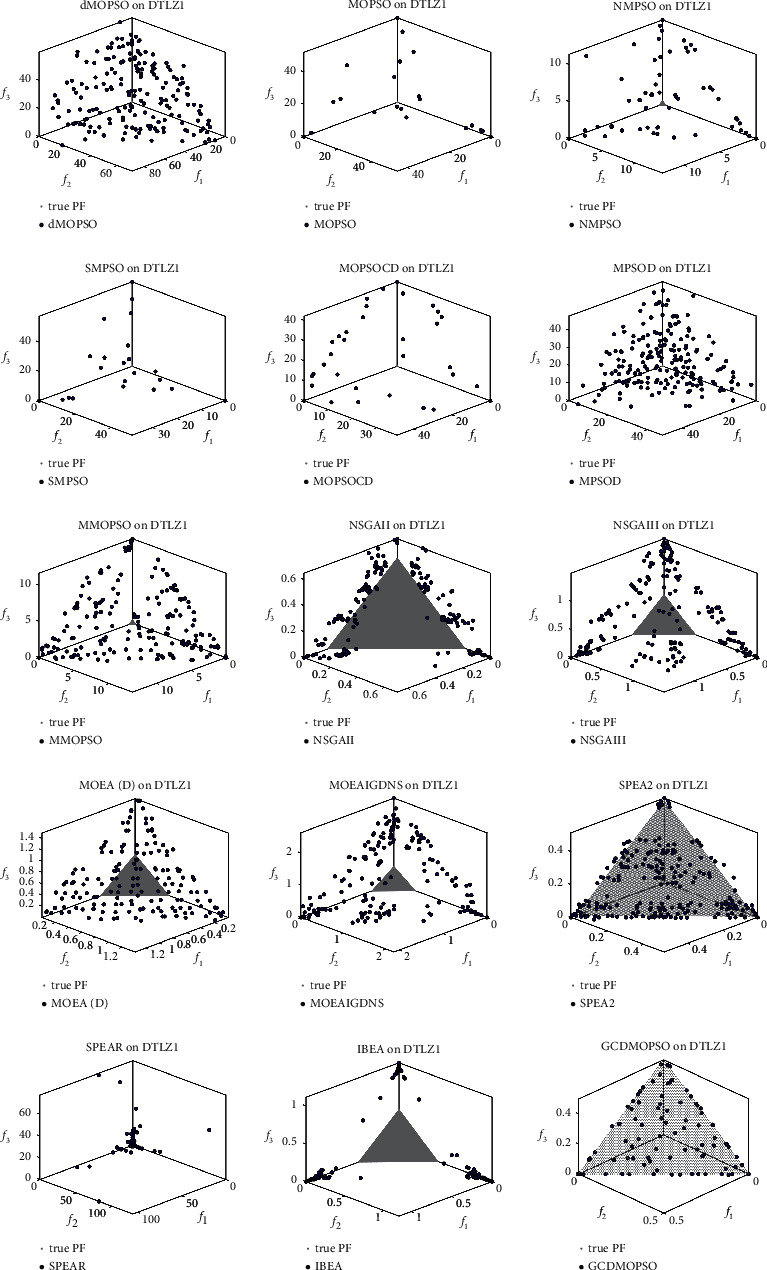
The nondominated set associated with the best IGD value among 30 runs obtained by the GCDMOPSO, and then compared MOPSOs and MOEAs on multiobjective DTLZ1.

As further observations, Figure 5 presents the nondominated set associated with the best IGD value among 30 runs obtained by the GCDMOPSO, and then MOPSOs and MOEAs were compared on multiobjective DTLZ1. The nondominated sets were obtained by dMOPSO, MOPSO, NMPSO, SMPSO, MOPSOCD, MPSO/D, MMOPSO, NSGA-II, NSGA-III, MOEA/D, MOEAIGDNS, SPEAR, SPEA2, IBEA, and GCDMOPSO, respectively. The experimental results showed that the proposed GCDMOPSO outperforms the compared MOPSOs and MOEAs in terms of both convergence and diversity on multiobjective DTLZ1.

### 3.3. Selection Strategy of Global Leader

**Figure 6:**
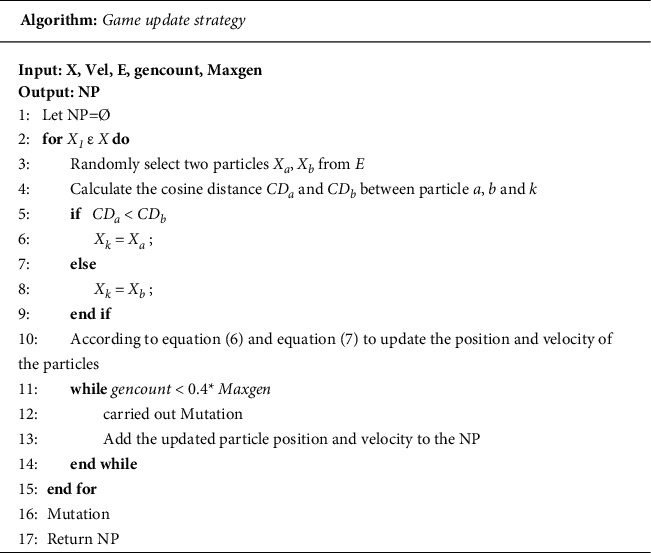
Game update strategy.

In MOPSOs, each individual has location information and velocity information, as well as the characteristics of information exchange between individuals. These individuals can learn from the best position in history (pbest) and the best position in the world (gbest) and then their position and velocity are updated through equations (6) and (7) in Section 2 to produce a new generation of groups. The choice of the global optimal position (gbest) is closely related to the distribution of nondominated solutions. If few dense nondominated solutions are distributed in a certain area, the sparsely distributed particles are more likely to become the global optimal particles. In order to strengthen the selection pressure of gbest, it was combined with the game update mechanism. Thus, a novel global optimal selection strategy of the game strategy was proposed. The original game group optimizer theory divides the original population into two parts: game success and game failure. The failed part of the game strategy learns from the successful part of the game, and the population is updated iteratively on this basis. References for the specific game process can be found in [30]. The game strategy proposed in this article is different from the original game mechanism. In this article, the game is played in the external archive, and the particles to be updated are randomly selected from two individuals in the external archive. The winner of the game will become the leader, guiding the failed individuals to search for the optimal set, and the successful individuals will maintain the original speed and direction. The specific update process is shown in Figure 6.

The game individuals in this game strategy were elected through nondominated sorting and grid optimal distance. The success or failure of the game is determined according to the cosine distance between the game individuals. The winner of the game acts as the global optimal individual to guide other individuals in the population to fly. In each pair of games, the individuals to be updated randomly select two nondominated solutions *a* and *b* from the external archive. The two nondominated solutions *a* and *b* are played through the cosine distance, and the game with a small cosine distance is successful. As shown in the pseudocode algorithm, the cosine distance between the nondominated solution *a* and individual *k* to be updated is small, so the nondominated solution *a* guides individual *k* to be updated to update the speed and position. The update formula is as follows:

(10) $$\begin{array}{c} v_{i}^{\prime }=c_{3}v_{i}+c_{4}\left(X_{k}-X_{i}\right),\\ X_{i}^{\prime }=X_{i}+v_{i}^{\prime }. \end{array}$$

In the above formula, *c*_3_ and *c*_4_ are randomly generated vectors between [0, 1], *X*_*k*_ is the position of the winner of the game, *X*_*i*_ is the current position of the particle, and *v*_*i*_ is the current velocity of the particle.

The whole process from selecting an external archive to comparing cosine distances is called game. Because the selected nondominated solution is random, the individual to be updated is not sure which guide will be selected in the end. The attributes of the leader will determine the effect of individual renewal, and the leader with better attributes will lead the update better. The effect of individual update depends on the leader entirely, so it is called game.

### 3.4. Steps of the GCDMOPSO

**Figure 7:**
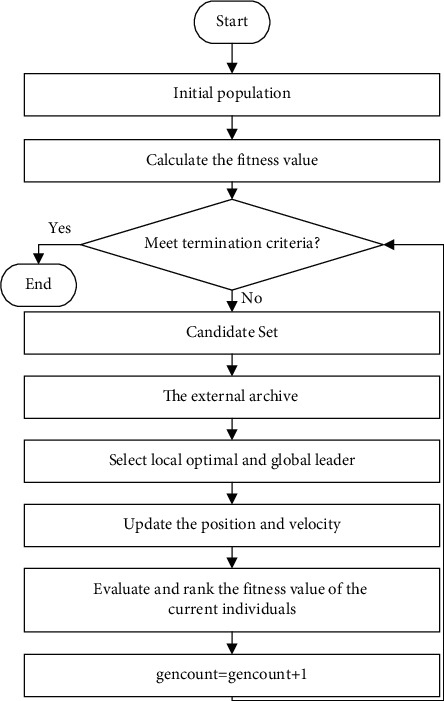
Main flow chart of the GCDMOPSO.

For MOPs, the objectives are mutually restricted. In MOPSOs, blindness is inevitable when controlling external archives and selecting the global optimum. This article proposes a novel strategy for external archive updates and global optimization. The main flow chart is shown in Figure 7 and the main steps of GCDMOPSO are as follows:

 
*Step 1*. The population was initialized, and acceleration constants *c*_1_ and *c*_2_ were set to guide other parameters.
 
*Step 2*. The fitness value of each individual was calculated, and nondominated sorting was performed by comparing its fitness value during the current iteration with the best historical fitness value.
 
*Step 3*. Whether the terminal conditions were met was determined. If met, output the results and terminate the algorithm. Otherwise, continue to the next step.
 
*Step 4*. A candidate set was created. By calculating the Euclidean distance from the origin of the coordinates to each individual, individuals with a shorter Euclidean distance were selected into the external archive.
 
*Step 5*. An external archive was created and the worst solution part of the external archive was deleted using the cosine distance measurement mechanism. At the same time, the candidate set was added as a storage mechanism for screened advantageous individuals' mechanism.
 
*Step 6*. The global optimal sample was selected. Using roulette and combining the game update mechanism, design a game strategy that incorporates the cosine distance measurement mechanism to select the global optimal sample.
 
*Step 7*. According to formulas (6) and (7), update the position and velocity.
 
*Step 8*. The fitness value of the current individuals was evaluated and ranked.
 
*Step 9*. gencount = gencount + 1 was set; then move to step 3.

## 4. Experimental Study

### 4.1. Test Problems

Comprehensive and diverse test problems were employed in order to assess the performance of GCDMOPSO. First, the ZDT test problems were adopted. If there are only the ZDT series of test functions, they are impossible to show the superior performance of GCDMOPSO. Therefore, other more difficult MOPs, the UF test problems, are used based on complex characteristics. In order to further test the performance of GCDMOPSO in processing MOPs with three objectives, DTLZ1–DTLZ5 and UF8–UF10 test problems are used in this article. These test problems cover most of the challenges in this area, such as many local Pareto fronts, convergence deviations, concavities, and discontinuities. The relevant settings of these test problems are given in Table 2.

Among them, *N* represents size of the population; *M* represents the number of objectives; *D* represents dimension of the decision variable; *FEs* represents the maximum number of evaluations. For fair comparison, all relevant parameters of the comparison algorithm are set according to the suggestions in the original reference. The population size *N* of two objectives and three objectives of each algorithm is set to 200, and the maximum number of fitness evaluations is fixed to 10000. For ZDT1–ZDT3 and all UF test problems, 30 decision variables are used, ZDT4 and ZDT6 used 10 decision variables, DTLZ1 used 7 decision variables, and DTLZ2–DTLZ5 used 12 decision variables. For ZDT1–ZDT3 and all UF test problems, 30 decision variables are used, ZDT4 and ZDT6 used 10 decision variables, DTLZ1 used 7 decision variables, and DTLZ2–DTLZ5 used 12 decision variables. In order to draw statistical conclusions, the number of independent runs of each test experiment is set to 30. For detailed information about ZDT, UF, and DTLZ test problems, the reader is referred to [31–33], respectively.

### 4.2. Performance Measures

The goal of MOPs is to find a uniformly distributed set that is as close to the true Pareto fronts as possible. In order to compare with other algorithms, this article uses inverted generation distance (IGD) [22] to evaluate the performance of GCDMOPSO. It is believed that this performance index can not only explain the convergence effects of the algorithm but also explain the distribution of the final solution. The true Pareto front for computing IGD was downloaded from http://jmetal.sourceforge.net/problems.html.

### 4.3. Experimental Settings

In the experiment, in order to verify the performance of GCDMOPSO in a convincing way, it was compared with seven state-of-the-art MOPSOs (i.e., dMOPSO [19], MOPSO [9], NMPSO [23], SMPSO [13], MOPSOCD [24], MPSO/D [18], and MMOPSO [14]) and seven classic MOEAs (i.e., NSGA-II [10], NSGA-III [25], MOEA/D [15], MOEAIGDNS [26], SPEAR [27], SPEA2 [28], and IBEA [29]), respectively. For fair comparison, all relevant parameters in the comparison algorithm are set according to their original references, as shown in Table 3. *p*_*c*_ and *p*_*m*_ are crossover probability and mutation probability in Table 3, respectively; *η*_*c*_ and *η*_*m*_ are the distribution indexes of SBX and PM, respectively; *F* and *CR* are parameters set by the authors in differential evolution; *T* is the number of divisions in genetic algorithm; div is the division network number of cells; *w*, *c*_1_, and *c*_2_ are the parameters of the velocity update equation used in the MOPSOs. The population size *N* of two objectives and three objectives of each algorithm is set to 200, and the maximum number of fitness evaluations is fixed to 10000; the size of the external file is set to be the same as *N*. In order to draw a statistical conclusion, the number of independent runs of each test experiment is set to 30. The average and standard deviation (std) on IGD are collected in corresponding Tables 4 and 5 for performance comparison. In addition, in order to determine the statistical significance, a Wilcoxon rank-sum test was further carried out to test the statistical significance of the difference between the results obtained by GCDMOPSO and the results obtained by other algorithms at *α* = 0.05. All experimental results are obtained on PC with 2.3 GHz CPU and 8 GB memory. All source codes of these competing algorithms are provided in the platform PlatEMO [34].

### 4.4. Comparisons of GCDMOPSO with Seven State-of-the-Art MOPSOs

In GCDMOPSO, seven MOPSOs and seven MOEAs are selected, and the program runs the average and standard deviation of the IGD values on ZDT1–ZDT4 and ZDT6, DTLZ1–DTLZ5, and UF1–UF10 in Table 4. Moreover, the Wilcoxon rank-sum test is adopted at a significance level of 0.05, where the symbols “+,” “−,” and “=” in the last row of the tables indicate that the result is significantly better than, significantly worse than, and statistically similar to that obtained by GCDMOPSO, respectively. The best average for each test instance is shown in bold.

It can be directly observed that the performance of the proposed GCDMOPSO is significantly better than the existing seven compared MOPSOs in terms of benchmark testing, that is, dMOPSO, MOPSO, NMPSO, SMPSO, MOPSOCD, MPSO/D, and MMOPSO. Of all 20 test instances, GCDMOPSO achieved statistically significantly better IGD values on 12 test instances which were far greater than those of the competing MOPSOs. For example, the numbers of optimal IGD values for dMOPSO, MOPSO, and MOPSOCD are zero, the number of optimal IGD values for MPSO/D is one, the numbers of optimal IGD values for NMPSO and SMPSO are two, and MMOPSO has five optimal IGD values.

**Figure 8:**
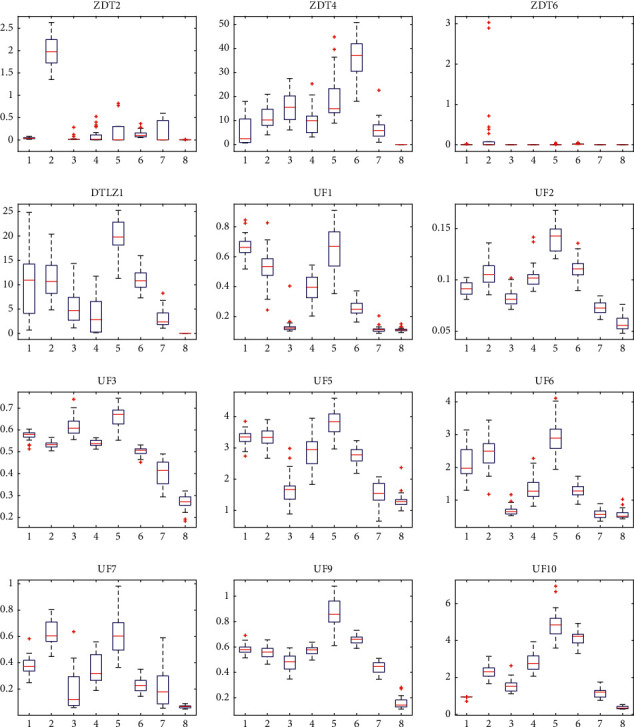
Statistical boxplot of IGD indicator of different MOPSOs and statistical boxplot of IGD indicator of different MOPSOs on ZDT2, ZDT4, ZDT6, DTLZ1, UF1–UF3, UF5–UF7, and UF9-UF10 problems, respectively.

For two-objective ZDT2, ZDT4, and ZDT6, the proposed GCDMOPSO can obtain a set of nondominant solutions, which can approximate the entire Pareto front well and maintain a good distribution. For the three-objective DTLZ1, the proposed GCDMOPSO can still achieve competitive performance, but, on the three-objective DTLZ2–DTLZ5, the performance of GCDMOPSO does not seem to be so ideal. It is worth noting that MMOPSO performed best on the two-objective ZDT1 and ZDT3, due to the fact that it has adopted the crossover and mutation operators in MOEAs in addition to the updating strategies of PSO. In UF1–UF10, the performance is far better than those of other comparison algorithms. Generally speaking, compared with the existing MOPSOs, the proposed GCDMOPSO proves the overall best performance. At the same time, when different algorithms are run independently 30 times, the partial statistical block diagram of the evaluation index IGD of GCDMOPSO algorithm and the comparison algorithm is shown in Figure 8 (1, 2, 3, 4, 5, 6, 7, and 8 represent dMOPSO, MOPSO, NMPSO, SMPSO, MOPSOCD, MPSO/D, MMOPSO, and GCDMOPSO, respectively). As shown in Figure 8, GCDMOPSO recorded the minimum values on ZDT2, ZDT4, ZDT6, DTLZ1, UF1–UF3, UF5–UF7, and UF9-UF10. It can be clearly seen from Figure 8 that GCDMOPSO can obtain better nondominated solutions compared with other MOPSOs. The results are consistent with the qualitative analysis in Table 4.

From the above empirical results, we can conclude that, compared with the existing MOPSOs, GCDMOPSO has application prospects in solving PSO.

### 4.5. Comparisons of GCDMOPSO with Seven Competitive MOEAs

Table 5 presents the mean and standard deviation of IGD values of NSGA-II, NSGA-III, MOEA/D, MOEAIGDNS, SPEAR, SPEA2, and IBEA on ZDT1 to ZDT4 and ZDT6, DTLZ1 to DTLZ5, and UF1 to UF10, where the Wilcoxon rank-sum test is also adopted and the best mean for each test instance is shown in bold. It can be observed that the performance of the proposed GCDMOPSO is significantly better than those of the seven compared MOEAs (i.e., NSGA-II, NSGA-III, MOEA/D, MOEAIGDNS, SPEAR, SPEA2, and IBEA) in terms of benchmark testing. According to the results, there are 12 test cases with statistically significant best performance among 20 test examples.

For two-objective ZDT1-ZDT2, ZDT4, and ZDT6, UF1–UF4, and UF7, GCDMOPSO performs best compared to the seven algorithms. For example, the numbers of optimal IGD values for NSGA-III and MOEAIGDNS are zero, the numbers of optimal IGD values for MOEA/D, SPEAR, and SPEA2 are one, the number of optimal IGD values for NSGA-II is three, and IBEA has four best IGD values. For the three-objective DTLZ series, compared MOEAs are obviously better than GCDMOPSO, and this is because genetic factors are more suitable for solving MOPs with local frontiers. Therefore, in the existing MOPs, more researchers suggest the main reason for using genetic factors.

**Figure 9:**
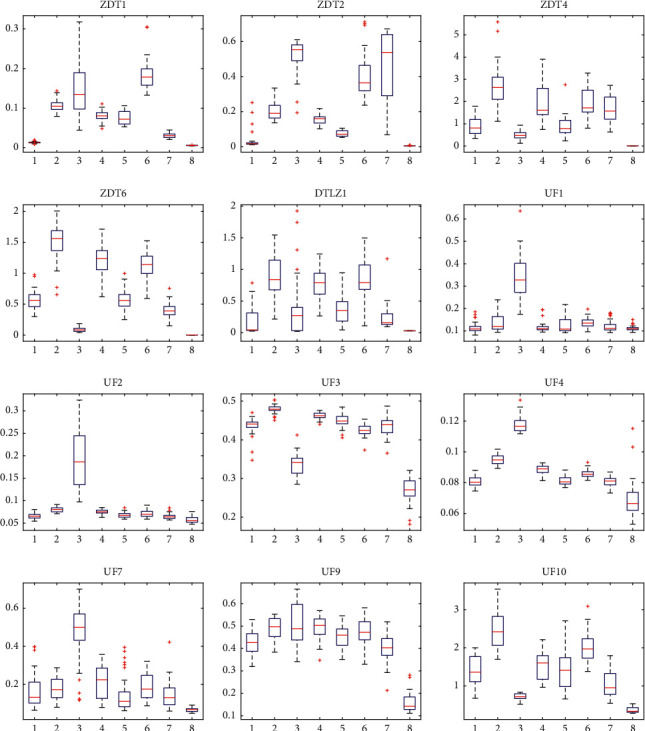
Statistical boxplot of IGD indicator of different MOEAs and statistical boxplot of IGD indicator of different MOEAs on ZDT1-ZDT2, ZDT4, ZDT6, DTLZ1, UF1–UF4, UF7, and UF9-UF10 problems, respectively.

At the same time, when different algorithms are run independently 30 times, the partial statistical block diagram of the evaluation index IGD of the GCDMOPSO and the comparison algorithm is shown in Figure 9 (1, 2, 3, 4, 5, 6, 7, and 8 represent NSGA-II, NSGA-III, MOEA/D, MOEAIGDNS, SPEAR, SPEA2, IBEA, and GCDMOPSO, respectively). As shown in Figure 9, GCDMOPSO recorded the minimum values on ZDT1, ZDT2, ZDT4, ZDT6, DTLZ1, UF1 to UF4, UF7, UF9, and UF10. It can be clearly seen from Figure 9 that GCDMOPSO can obtain best nondominated solutions compared with other MOEAs. The results are consistent with the qualitative analysis in Table 5.

From the above empirical results, we can conclude that, compared with the existing MOEAs, GCDMOPSO has application prospects in solving PSO.

### 4.6. Complexity of the GCDMOPSO

The complexity of the proposed GCDMOPSO depends on the complexity of its components, that is, the complexity of game strategy and cosine distance. The following is the complexity analysis of GCDMOPSO.

Suppose that the population size is *N*, where there are *m* nondominated individuals. In general, it is assumed that *k*(*m* ≤ *k* ≤ *N*) games have been played. According to the game strategy, an individual will be eliminated after each game. Therefore, a total of *k* individuals were eliminated, including *q*(0 < *q* ≤ *m*) dominated individuals and *p*(0 ≤ *p* < *N* − *m*) nondominated individuals. After *k* times of games, (*N* − *k*) individuals become winners among the *N* individuals. For the convenience of analysis, assuming that, in each game, (*N* − *k*) dominated individuals have the same probability of being selected, after *k* games, there are

(11) $$\begin{array}{c} T\left(n\right)=\left(N-1\right)+\left[\left(N-2\right)-\frac{\left(N-k\right)}{k}\right]+\left[\left(N-3\right)-\frac{2\left(N-k\right)}{k}\right]+\cdots +\left[\left(N-k\right)-\frac{\left(k-1\right)\left(N-k\right)}{k}\right]\\ =\left[\left(\left(N-1\right)+\left(N-k\right)\right)\frac{k}{2}-\left(\frac{\left(N-k\right)}{k}+\frac{k\left(N-k\right)}{k}\right)\frac{k}{2}\right]+\left(N-k\right)\\ =\left(N-1\right)\frac{k}{2}+\left(N-k\right)\frac{1}{2}\leq N^{2}. \end{array}$$

The time complexity of the game strategy is *T*(*n*)=*O*(*N*^2^). At the same time, in the process of updating the external archive of the game strategy, the calculation complexity of the cosine distance is *O*(*M* × (2*N*)log_2_(2*N*)). Then the total computational complexity is *O*(*M* × *N*^2^), where *M* is the number of objectives.

In addition, this article uses MATLAB functions (tic and toc) to calculate the runtime (unit: second) of each algorithm when the number of evaluations is 10000. It can be seen from Tables 6 and 7 that even though GCDMOPSO uses the cosine distance measurement mechanism and the game strategy, the time complexity of GCDMOPSO and other comparison algorithms is on the same order of magnitude on functions ZDT1–ZDT4 and ZDT6, DTLZ1–DTLZ5, and UF1–UF10.

## 5. Conclusions

This paper has proposed a novel multiobjective particle swarm optimization based on cosine distance mechanism and game strategy to solve MOPs. The optimization was used to update the Pareto set in the external archives through the update strategy of the cosine distance measurement mechanism and add a candidate set as a storage for screened advantageous individuals' mechanism. The optimization is conducive to Pareto optimal set close to the true Pareto optimal front and maintains the diversity of the swarm. In order to improve the performance of optimization, this article combined the game update strategy to design a global optimal selection strategy of the game strategy based on the cosine distance measurement mechanism. These experimental studies have shown that the proposed GCDMOPSO has better performance than several state-of-the-art MOPSOs and competitive MOEAs.

## Figures and Tables

**Table 1:** List of acronyms.

Acronyms	The full name of an acronym
MOPs [1]	Multiobjective optimization problems
PSO [7]	Particle swarm optimization
MOPSOs	Multiobjective particle swarm optimization algorithms
MOEAs	Multiobjective evolutionary algorithms
GCDMOPSO	Multiobjective particle swarm optimization based on cosine distance mechanism and game strategy
MOPSO [9]	Handling multiple objectives with particle swarm optimization
NSGA-II [10]	A fast and elitist multiobjective genetic algorithm
PAES [11]	Approximating the nondominated front using the Pareto archived evolution strategy
SMPSO [13]	A new PSO-based metaheuristic for multiobjective optimization
MMOPSO [14]	A novel multiobjective particle swarm optimization with multiple search strategies
MOEA/D [15]	A multiobjective evolutionary algorithm based on decomposition
SDMOPSO [17]	A novel smart multiobjective particle swarm optimization using decomposition
dMOPSO [19]	A multiobjective particle swarm optimizer based on decomposition
MOPSONN [20]	A fast multiobjective particle swarm optimization algorithm based on a new archive updating mechanism
IGD [22]	Inverted generational distance
NMPSO [23]	Particle swarm optimization with a balance able fitness estimation for many-objective optimization problems
MOPSOCD [24]	An effective use of crowding distance in multiobjective particle swarm optimization
MPSO/D [18]	A new multiobjective particle swarm optimization algorithm based on decomposition
NSGA-III [25]	An evolutionary many-objective optimization algorithm using reference point-based nondominated sorting approach, part I: solving problems with box constraints
MOEAIGDNS [26]	A multiobjective evolutionary algorithm based on an enhanced inverted generational distance metric
SPEAR [27]	A strength Pareto evolutionary algorithm based on reference direction for multiobjective and many-objective optimization
SPEA2 [28]	Improving the strength Pareto evolutionary algorithm
IBEA [29]	Indicator-based selection in multiobjective search
*N*	The population size
*M*	The number of objectives
*D*	Dimension of the decision variable
*FEs*	The maximum number of evaluations
*p*_*c*_	Crossover probability
*p*_*m*_	Mutation probability
SBX	Simulated binary crossover
PM	Polynomial-based mutation
*η*_*c*_	The distribution indexes of SBX
*η*_*m*_	The distribution indexes of PM
*F*	Parameters set by the author in differential evolution
*CR*	Parameters set by the author in differential evolution
div	The division network number of cells
pbest	Personal best particle
gbest	Global best particle

**Table 2:** Population size, number of objectives, dimensions, and the maximum number of evaluations of the chosen test problems.

Problems	*N*	*M*	*D*	*FEs*
ZDT1–ZDT3	200	2	30	10000
ZDT4 and ZDT6	200	2	10	10000
DTLZ1	200	3	7	10000
DTLZ2–DTLZ5	200	3	12	10000
UF1–UF7	200	2	30	10000
UF8–UF10	200	3	30	10000

**Table 3:** Parameters settings of GCDMOPSO and all the compared algorithms.

	Algorithms	Parameters setting
1	dMOPSO [19]	*w* ∈ [0.1, 0.5], *c*_1_*c*_2_ ∈ [1.5, 2.5]
2	MOPSO [9]	*w* ∈ [0.1, 0.5], *c*_1_*c*_2_ ∈ [1.5, 2.5], div=10
3	SMPSO [13]	*w* ∈ [0.1, 0.5], *c*_1_*c*_2_ ∈ [1.5, 2.5], *p*_*m*_=(1/*n*), *η*_*m*_=20
4	MOPSOCD [24]	*w* ∈ [0.1, 0.5], *c*_1_*c*_2_ ∈ [1.5, 2.5]
5	MPSO/D [18]	*w* ∈ [0.1, 0.5], *c*_1_*c*_2_ ∈ [1.5, 2.5], *p*_*c*_=0.9, *F*=0.5, *CR*=0.5, *p*_*m*_=(1/*n*), *η*_*m*_=20
6	MMOPSO [14]	*w* ∈ [0.1, 0.5], *c*_1_*c*_2_ ∈ [1.5, 2.5], *p*_*c*_=0.9, *p*_*m*_=(1/*n*), *η*_*c*_=*η*_*m*_=20
7	NMPSO [23]	*w* ∈ [0.1, 0.5], *c*_1_*c*_2_*c*_3_ ∈ [1.5, 2.5], *p*_*m*_=(1/*n*), *η*_*c*_=*η*_*m*_=20
8	NSGA-II [10]	*p*_*c*_=1.0, *p*_*m*_=(1/*n*), *η*_*c*_=*η*_*m*_=20
9	NSGA-III [25]	*p*_*c*_=1.0, *p*_*m*_=(1/*n*), *η*_*c*_=*η*_*m*_=20
10	MOEA/D [15]	*p*_*c*_=1.0, *p*_*m*_=(1/*n*), *η*_*c*_=*η*_*m*_=20, *T*=20
11	MOEAIGDNS [26]	*p*_*c*_=1.0, *p*_*m*_=(1/*n*), *η*_*c*_=*η*_*m*_=20
12	SPEAR [27]	*p*_*c*_=1.0, *p*_*m*_=(1/*n*), *η*_*c*_=*η*_*m*_=20
13	SPEA2 [28]	*p*_*c*_=1.0, *p*_*m*_=(1/*n*), *η*_*c*_=*η*_*m*_=20
14	IBEA [29]	*p*_*c*_=1.0, *p*_*m*_=(1/*n*), *η*_*c*_=*η*_*m*_=20
15	GCDMOPSO	*w*=0.4, *c*_1_*c*_2_=2, div=50

**Table 4:** IGD values of the proposed GCDMOPSO and seven MOPSOs on ZDT1–ZDT4 and ZDT6, DTLZ1–DTLZ5, and UF1–UF10 test problems.

Problems	dMOPSO	MOPSO	NMPSO	SMPSO	MOPSOCD	MPSO/D	MMOPSO	GCDMOPSO
ZDT1	5.3199*e* − 2 (1.84*e* − 2) −	1.2818*e* + 0 (1.61*e* − 1) −	3.5090*e* − 2 (2.49*e* − 2) −	7.8490*e* − 2 (8.12*e* − 2) −	1.2410*e* − 2 (3.41*e* − 2) −	1.0097*e* − 1 (3.75*e* − 2) −	**2.4320e − 3 (9.90e − 5)** **+**	5.5096*e* − 3 (5.47*e* − 4)
ZDT2	4.0278*e* − 2 (1.65*e* − 2) −	1.9587*e* + 0 (3.14*e* − 1) −	3.2220*e* − 2 (5.23*e* − 2) −	8.8623*e* − 2 (1.40*e* − 1) −	1.3199*e* − 1 (2.20*e* − 1) −	1.3635*e* − 1 (9.22*e* − 2) −	1.8831*e* − 1 (2.43*e* − 1) −	**5.9683e − 3 (3.86e − 5)**
ZDT3	3.6856*e* − 2 (1.09*e* − 2) +	7.9559*e* − 1 (1.88*e* − 1) −	9.3162*e* − 2 (1.80*e* − 2) +	1.9286*e* − 1 (8.21*e* − 2) =	5.2211*e* − 2 (6.74*e* − 2) +	1.9101*e* − 1 (5.42*e* − 2) =	**5.4929e − 3 (1.51e − 2)** **+**	2.0214*e* − 1 (4.10*e* − 3)
ZDT4	5.6682*e* + 0 (6.02*e* + 0) −	1.0959*e* + 1 (4.09*e* + 0) −	1.5558*e* + 1 (6.00*e* + 0) −	9.6916*e* + 0 (5.27*e* + 0) −	1.9321*e* + 1 (9.00*e* + 0) −	3.6610*e* + 1 (6.82*e* + 0) −	6.4024*e* + 0 (3.87*e* + 0) −	**5.1106e − 3 (5.31e − 4)**
ZDT6	4.7132*e* − 3 (5.34*e* − 3) −	2.6553*e* − 1 (7.51*e* − 1) −	2.2710*e* − 3 (1.83*e* − 4) −	1.9179*e* − 3 (6.27*e* − 5) =	5.6155*e* − 3 (8.77*e* − 3) −	1.7602*e* − 2 (1.00*e* − 2) −	2.0980*e* − 3 (9.98*e* − 5) =	**1.9036e − 3 (2.19E − 4)**
DTLZ1	1.0110*e* + 1 (6.03*e* + 0) −	1.1068*e* + 1 (3.98*e* + 0) −	5.3123*e* + 0 (3.22*e* + 0) −	3.7308*e* + 0 (3.56*e* + 0) −	1.9868*e* + 1 (3.43*e* + 0) −	1.0204*e* + 1 (2.84*e* + 0) −	2.6475*e* + 0 (2.13*e* + 0) −	**3.1893e − 2 (1.60e − 3)**
DTLZ2	1.0357*e* − 1 (4.89*e* − 3) −	9.9168*e* − 2 (1.83*e* − 2) −	5.5695*e* − 2 (1.66*e* − 3) −	6.2961*e* − 2 (5.67*e* − 3) +	9.6187*e* − 2 (1.07*e* − 2) +	**4.5104e − 2 (1.27e − 3)** **+**	5.1981*e* − 2 (1.22*e* − 3) +	1.3156*e* − 1 (1.24*e* − 2)
DTLZ3	9.5459*e* + 1 (7.27*e* + 1) +	1.8585*e* + 2 (4.40*e* + 1) =	1.1797*e* + 2 (2.45*e* + 1) =	**4.2214e + 1 (4.28e + 1) +**	1.2284*e* + 2 (4.00*e* + 1) =	1.4031*e* + 2 (1.58*e* + 1) =	9.6313*e* + 1 (2.83*e* + 1) +	1.2848*e* + 2 (4.16*e* + 1)
DTLZ4	3.3629*e* − 1 (2.32*e* − 2) −	3.5379*e* − 1 (1.02*e* − 1) −	**5.7723e − 2 (1.48e − 3)** **+**	4.0061*e* − 1 (1.22*e* − 1) −	3.2468*e* − 1 (4.23*e* − 2) −	1.3759*e* − 1 (3.72*e* − 2) =	8.0754*e* − 2 (1.63*e* − 1) +	2.1915*e* − 1 (5.23−2)
DTLZ5	2.7069*e* − 2 (4.04*e* − 3) =	7.3854*e* − 3 (1.46*e* − 3) +	7.1965*e* − 3 (1.09*e* − 3) +	**3.5139e − 3 (3.37e − 4) +**	3.5753*e* − 2 (1.28*e* − 2) =	5.5742*e* − 2 (6.20*e* − 3) −	3.6474*e* − 3 (3.25*e* − 4) +	3.0289*e* − 2 (5.98*e* − 3)
UF1	6.6464*e* − 1 (7.81*e* − 2) −	5.2791*e* − 1 (1.19*e* − 1) −	1.3296*e* − 1 (5.34*e* − 2) =	3.9727*e* − 1 (8.95*e* − 2) −	6.5297*e* − 1 (1.48*e* − 1) −	2.6644*e* − 1 (4.60*e* − 2) −	1.2561*e* − 1 (6.35*e* − 2) =	**1.1136e − 1 (1.20e − 2)**
UF2	9.1522*e* − 2 (6.36*e* − 3) −	1.0596*e* − 1 (1.30*e* − 2) −	8.2356*e* − 2 (6.97*e* − 3) −	1.0320*e* − 1 (1.21*e* − 2) −	1.4038*e* − 1 (1.37*e* − 2) −	1.1472*e* − 1 (6.45*e* − 3) −	7.0445*e* − 2 (6.81*e* − 3) −	**5.7949e − 2 (6.63e − 3)**
UF3	5.7528*e* − 1 (2.11*e* − 2) −	5.3232*e* − 1 (1.45*e* − 2) −	6.1950*e* − 1 (4.55*e* − 2) −	5.3918*e* − 1 (1.35*e* − 2) −	6.6073*e* − 1 (4.87*e* − 2) −	6.7669*e* − 1 (2.46*e* − 2) −	5.6722*e* − 1 (1.45*e* − 2) −	**2.6948e − 1 (3.46e − 2)**
UF4	1.3762*e* − 1 (5.27*e* − 3) −	1.1569*e* − 1 (1.17*e* − 2) −	6.2625*e* − 2 (9.38*e* − 3) =	1.1333*e* − 1 (7.72*e* − 3) −	7.8445*e* − 2 (7.46*e* − 3) −	9.7497*e* − 2 (5.79*e* − 3) −	**5.6336e − 2 (3.49e − 3)** **+**	6.9376*e* − 2 (1.27*e* − 2)
UF5	3.3212*e* + 0 (2.49*e* − 1) −	3.3737*e* + 0 (2.78*e* − 1) −	1.6778*e* + 0 (5.00*e* − 1) −	2.8624*e* + 0 (5.32*e* − 1) −	3.7922*e* + 0 (3.91*e* − 1) −	2.7759*e* + 0 (2.63*e* − 1) −	1.5025*e* + 0 (3.04*e* − 1) =	**1.3195e + 0 (2.50e − 1)**
UF6	2.1692*e* + 0 (5.25*e* − 1) −	2.4512*e* + 0 (4.97*e* − 1) −	6.9450*e* − 1 (1.55*e* − 1) −	1.3552*e* + 0 (3.86*e* − 1) −	2.8954*e* + 0 (5.38*e* − 1) −	1.4377*e* + 0 (2.33*e* − 1) −	5.7258*e* − 1 (1.09*e* − 1) =	**5.7057e − 1 (1.33e − 1)**
UF7	3.7871*e* − 1 (6.98*e* − 2) −	6.2837*e* − 1 (9.78*e* − 2) −	1.8881*e* − 1 (1.44*e* − 1) −	3.5724*e* − 1 (1.15*e* − 1) −	6.0699*e* − 1 (1.35*e* − 1) −	2.5060*e* − 1 (7.27*e* − 2) −	1.1800*e* − 1 (8.76*e* − 2) −	**6.6068e − 2 (9.87e − 3)**
UF8	3.4764*e* − 1 (4.67*e* − 2) =	4.2142*e* − 1 (3.93*e* − 2) −	4.7503*e* − 1 (1.03*e* − 1) −	3.9131*e* − 1 (4.44*e* − 2) =	8.7353*e* − 1 (1.71*e* − 1) −	5.5120*e* − 1 (5.13*e* − 2) −	**2.6889e − 1 (1.21e − 2)** **=**	3.8469*e* − 1 (7.12*e* − 2)
UF9	5.8445*e* − 1 (3.75*e* − 2) −	5.5568*e* − 1 (4.36*e* − 2) −	4.7656*e* − 1 (6.25*e* − 2) −	5.7107*e* − 1 (3.58*e* − 2) −	8.6673*e* − 1 (1.19*e* − 1) −	6.5999*e* − 1 (3.74*e* − 2) −	4.3376*e* − 1 (5.09*e* − 2) −	**1.5833e − 1 (4.39e − 2)**
UF10	9.3743*e* − 1 (4.31*e* − 2) −	2.2931*e* + 0 (3.26*e* − 1) −	1.5264*e* + 0 (3.37*e* − 1) −	2.7945*e* + 0 (4.45*e* − 1) −	4.8780*e* + 0 (7.67*e* − 1) −	4.1813*e* + 0 (3.21*e* − 1) −	1.2502*e* + 0 (3.77*e* − 1) −	**3.7067e − 1 (7.55e − 2)**
+/−/=	2/16/2	1/18/1	3/14/3	3/14/3	3/16/1	1/16/3	6/8/6	—
Best/all	0/20	0/20	1/20	2/20	0/20	1/20	4/20	12/20

**Table 5:** IGD values of the proposed GCDMOPSO and seven MOEAs on ZDT1–ZDT4 and ZDT6, DTLZ1–DTLZ5, and UF1–UF10 test problems.

Problems	NSGA-II	NSGA-III	MOEA/D	MOEAIGDNS	SPEAR	SPEA2	IBEA	GCDMOPSO
ZDT1	1.3351*e* − 2 (2.24*e* − 3) −	1.0794*e* − 1 (1.64*e* − 2) −	1.4869*e* − 1 (7.18*e* − 2) −	8.0204*e* − 2 (1.34*e* − 2) −	1.8544*e* − 1 (4.17*e* − 2) −	4.5791*e* − 2 (1.02*e* − 2) −	3.0575*e* − 2 (6.09*e* − 3) −	**5.5096e − 3 (5.47e − 4)**
ZDT2	3.7065*e* − 2 (5.67*e* − 2) −	2.0316*e* − 1 (4.74*e* − 2) −	5.1303*e* − 1 (1.03*e* − 1) −	1.5661*e* − 1 (2.61*e* − 2) −	4.0898*e* − 1 (1.29*e* − 1) −	7.5782*e* − 2 (1.76*e* − 2) −	4.5806*e* − 1 (2.25*e* − 1) −	**5.9683e − 3 (1.00e − 3)**
ZDT3	**1.4048e − 2 (9.19e − 3)** **+**	9.3239*e* − 2 (1.33*e* − 2) +	1.5966*e* − 1 (4.04*e* − 2) =	6.5803*e* − 2 (1.32*e* − 2) +	1.5273*e* − 1 (1.70*e* − 2) =	3.8329*e* − 2 (9.18*e* − 3) +	2.4122*e* − 2 (5.68*e* − 3) +	2.0214*e* − 1 (4.10*e* − 3)
ZDT4	9.1119*e* − 1 (3.95*e* − 1) −	2.7254*e* + 0 (9.71*e* − 1) −	4.7901*e* − 1 (1.92*e* − 1) −	1.9498*e* + 0 (8.48*e* − 1) −	1.9591*e* + 0 (6.21*e* − 1) −	8.7718*e* − 1 (4.89*e* − 1) −	1.6959*e* + 0 (5.78*e* − 1) −	**5.1106e − 3 (5.31e − 4)**
ZDT6	5.7138*e* − 1 (1.55*e* − 1) −	1.5068*e* + 0 (3.09*e* − 1) −	8.9624*e* − 2 (3.52*e* − 2) −	1.2194*e* + 0 (2.58*e* − 1) −	1.1220*e* + 0 (2.14*e* − 1) −	5.8304*e* − 1 (1.80*e* − 1) −	4.0693*e* − 1 (1.37*e* − 1) −	**1.9036e − 3 (2.19E − 4)**
DTLZ1	1.9064*e* − 1 (2.17*e* − 1) −	8.7852*e* − 1 (3.52*e* − 1) −	4.0238*e* − 1 (5.09*e* − 1) −	7.5255*e* − 1 (2.57*e* − 1) −	8.5648*e* − 1 (3.23*e* − 1) −	3.7942*e* − 1 (2.38*e* − 1) −	2.5592*e* − 1 (2.18*e* − 1) −	**3.1893e − 2 (1.60e − 3)**
DTLZ2	5.0991*e* − 2 (1.25*e* − 3) +	4.0110*e* − 2 (6.49*e* − 4) +	**3.9672e − 2 (8.95e − 4)** **+**	4.3567*e* − 2 (1.45*e* − 3) +	4.2995*e* − 2 (1.69*e* − 3) +	3.9796*e* − 2 (5.62*e* − 4) +	6.0008*e* − 2 (1.88*e* − 3) +	1.3156*e* − 1 (1.24*e* − 2)
DTLZ3	1.5011*e* + 1 (5.61*e* + 0) +	3.3597*e* + 1 (7.77*e* + 0) +	1.9932*e* + 1 (1.05*e* + 1) +	3.0896*e* + 1 (8.95*e* + 0) +	3.5538*e* + 1 (8.93*e* + 0) +	1.4161*e* + 1 (4.45*e* + 0) +	**1.1652e + 1 (4.42e + 0) +**	1.2848*e* + 2 (4.16*e* + 1)
DTLZ4	6.7625*e* − 2 (8.95*e* − 2) +	5.7588*e* − 2 (9.14*e* − 2) =	3.7432*e* − 1 (3.48*e* − 1) =	6.0124*e* − 2 (9.09*e* − 2) +	**4.4537e − 2 (1.19e − 3)** **+**	7.4018*e* − 2 (1.27*e* − 1) +	5.8439*e* − 2 (1.48*e* − 3) +	2.1915*e* − 1 (5.23−2)
DTLZ5	**3.9962e − 3 (2.48e − 4)** **+**	7.1312*e* − 3 (9.16*e* − 4) +	2.0348*e* − 2 (6.23*e* − 4) +	5.6171*e* − 3 (7.32*e* − 4) +	2.2074*e* − 2 (1.56*e* − 3) =	4.5566*e* − 3 (5.73*e* − 4) +	1.1354*e* − 2 (9.22*e* − 4) +	3.0289*e* − 2 (5.98*e* − 3)
UF1	1.1632*e* − 1 (2.49*e* − 2) −	1.4092*e* − 1 (4.21*e* − 2) −	3.3299*e* − 1 (1.01*e* − 1) −	1.1721*e* − 1 (2.51*e* − 2) −	1.3790*e* − 1 (2.30*e* − 2) −	1.2188*e* − 1 (3.16*e* − 2) −	1.2150*e* − 1 (2.75*e* − 2) −	**1.1136e − 1 (1.20e − 2)**
UF2	6.5645*e* − 2 (6.00*e* − 3) −	8.0437*e* − 2 (5.54*e* − 3) −	1.8876*e* − 1 (6.80*e* − 2) −	7.5721*e* − 2 (4.95*e* − 3) −	7.0807*e* − 2 (8.07*e* − 3) −	6.8572*e* − 2 (6.20*e* − 3) −	6.5305*e* − 2 (6.26*e* − 3) −	**5.7949e − 2 (6.63e − 3)**
UF3	4.3498*e* − 1 (2.51*e* − 2) −	4.7938*e* − 1 (1.13*e* − 2) −	3.3698*e* − 1 (2.81*e* − 2) −	4.6226*e* − 1 (8.68*e* − 3) −	4.2464*e* − 1 (1.60*e* − 2) −	4.4909*e* − 1 (1.74*e* − 2) −	4.3557*e* − 1 (2.52*e* − 2) −	**2.6948e − 1 (3.46e − 2)**
UF4	8.0553*e* − 2 (3.46*e* − 3) −	9.4935*e* − 2 (3.23*e* − 3) −	1.1790*e* − 1 (5.10*e* − 3) −	8.8455*e* − 2 (2.99*e* − 3) −	8.5693*e* − 2 (2.53*e* − 3) −	8.0996*e* − 2 (2.55*e* − 3) −	8.0710*e* − 2 (2.88*e* − 3) −	**6.9376e − 2 (1.27e − 2)**
UF5	7.8265*e* − 1 (2.68*e* − 1) +	1.6238*e* + 0 (3.91*e* − 1) −	1.3193*e* + 0 (2.96*e* − 1) =	1.0254*e* + 0 (2.49*e* − 1) =	1.2043*e* + 0 (2.25*e* − 1) =	9.3783*e* − 1 (2.90*e* − 1) +	**7.4200e − 1 (1.62e − 1)** **+**	1.3195*e* + 0 (2.50*e* − 1)
UF6	5.3210*e* − 1 (8.41*e* − 2) =	7.8385*e* − 1 (1.85*e* − 1) −	5.9812*e* − 1 (2.41*e* − 1) −	6.0366*e* − 1 (1.20*e* − 1) −	6.8918*e* − 1 (1.02*e* − 1) −	5.1892*e* − 1 (8.12*e* − 2) =	**5.1057e − 1 (9.97e − 2)** **=**	5.7057*e* − 1 (1.33*e* − 1)
UF7	1.7024*e* − 1 (9.95*e* − 2) −	1.7604*e* − 1 (6.34*e* − 2) −	4.6220*e* − 1 (1.73*e* − 1) −	2.1070*e* − 1 (9.19*e* − 2) −	1.8685*e* − 1 (7.08*e* − 2) −	1.5383*e* − 1 (9.99*e* − 2) −	1.4710*e* − 1 (7.88*e* − 2) −	**6.6068e − 2 (9.87e − 3)**
UF8	3.1419*e* − 1 (3.67*e* − 2) =	3.3684*e* − 1 (3.82*e* − 2) =	5.7398*e* − 1 (2.50*e* − 1) −	3.7209*e* − 1 (4.31*e* − 2) =	3.1745*e* − 1 (2.05*e* − 2) +	**3.0404e − 1 (3.44e − 2)** **+**	3.7170*e* − 1 (5.25*e* − 2) =	3.8469*e* − 1 (7.12*e* − 2)
UF9	4.3302*e* − 1 (5.11*e* − 2) −	4.9207*e* − 1 (4.63*e* − 2) −	5.0362*e* − 1 (9.59*e* − 2) −	4.9717*e* − 1 (5.14*e* − 2) −	4.7406*e* − 1 (5.61*e* − 2) −	4.4935*e* − 1 (5.18*e* − 2) −	4.0120*e* − 1 (6.62*e* − 2) −	**1.5833e − 1 (4.39e − 2)**
UF10	1.3928*e* + 0 (4.05*e* − 1) −	2.4384*e* + 0 (4.75*e* − 1) −	7.1987*e* − 1 (8.22*e* − 2) −	1.5321*e* + 0 (3.69*e* − 1) −	2.0141*e* + 0 (4.27*e* − 1) −	1.4443*e* + 0 (5.10*e* − 1) −	1.0332*e* + 0 (3.23*e* − 1) −	**3.7067e − 1 (7.55e − 2)**
+/−/=	6/12/2	4/14/2	3/14/3	5/13/2	4/13/3	7/12/1	6/12/2	—
Best/all	2/20	0/20	1/20	0/20	1/20	1/20	3/20	12/20

**Table 6:** Runtime of different MOPSOs and GCDMOPSO on ZDT1–ZDT4 and ZDT6, DTLZ1–DTLZ5, and UF1–UF10 problems, respectively.

Problems	*FEs*	dMOPSO	MOPSO	NMPSO	SMPSO	MOPSOCD	MPSOD	MMOPSO	GCDMOPSO
ZDT1	10000	7.8676*e* − 1	3.1199*e* − 1	1.5439*e* + 1	3.0488*e* − 1	3.4421*e* − 1	1.8922*e* + 0	3.7923*e* − 1	4.2437*e* + 0
ZDT2	10000	6.8131*e* − 1	2.6821*e* − 1	1.1491*e* + 1	2.3180*e* − 1	3.7589*e* − 1	1.5781*e* + 0	3.1551*e* − 1	4.7586*e* + 0
ZDT3	10000	7.8204*e* − 1	2.6363*e* − 1	1.1106*e* + 1	2.3377*e* − 1	2.1194*e* − 1	1.5873*e* + 0	3.2188*e* − 1	3.2039*e* + 0
ZDT4	10000	8.7783*e* − 1	2.4611*e* − 1	2.2032*e* + 0	2.0048*e* − 1	1.7586*e* − 1	1.0354*e* + 0	2.4560*e* − 1	4.3560*e* + 0
ZDT6	10000	6.8091*e* − 1	2.9741*e* − 1	1.8178*e* + 1	2.0287*e* − 1	3.3332*e* − 1	1.2271*e* + 0	2.8039*e* − 1	4.3096*e* + 0
DTLZ1	10000	9.7676*e* − 1	2.5560*e* − 1	3.6316*e* + 0	1.9261*e* − 1	1.8182*e* − 1	2.1156*e* + 0	3.3008*e* − 1	2.7750*e* + 0
DTLZ2	10000	8.6479*e* − 1	5.5054*e* − 1	1.3995*e* + 1	3.4870*e* − 1	2.3072*e* − 1	2.0819*e* + 0	4.0736*e* − 1	3.1383*e* + 0
DTLZ3	10000	9.2875*e* − 1	2.4352*e* − 1	2.6860*e* + 0	2.1345*e* − 1	1.8944*e* − 1	2.1073*e* + 0	2.7857*e* − 1	1.5740*e* + 0
DTLZ4	10000	8.7179*e* − 1	3.3241*e* − 1	1.3662*e* + 1	2.5100*e* − 1	1.8809*e* − 1	1.4432*e* + 0	4.0681*e* − 1	0.8514*e* + 0
DTLZ5	10000	8.8027*e* − 1	4.6302*e* − 1	1.0695*e* + 1	3.5398*e* − 1	2.0460*e* − 1	1.4525*e* + 0	4.0731*e* − 1	1.7879*e* + 0
UF1	10000	8.3878*e* − 1	2.7608*e* − 1	2.2603*e* + 0	2.3215*e* − 1	2.1324*e* − 1	1.8870*e* + 0	3.2098*e* − 1	1.0027*e* + 0
UF2	10000	9.0479*e* − 1	2.9694*e* − 1	3.5757*e* + 0	2.5161*e* − 1	2.2116*e* − 1	1.9781*e* + 0	3.4488*e* − 1	1.7896*e* + 0
UF3	10000	8.4054*e* − 1	2.9903*e* − 1	5.4222*e* + 0	2.4683*e* − 1	2.2645*e* − 1	1.8899*e* + 0	3.6710*e* − 1	1.3945*e* + 0
UF4	10000	9.1935*e* − 1	2.7600*e* − 1	8.3688*e* + 0	2.4176*e* − 1	2.2351*e* − 1	2.0474*e* + 0	3.3159*e* − 1	1.7119*e* + 0
UF5	10000	9.3696*e* − 1	2.6467*e* − 1	2.1922*e* + 0	2.3675*e* − 1	2.1536*e* − 1	1.7893*e* + 0	3.3005*e* − 1	7.5826*e* − 1
UF6	10000	9.1887*e* − 1	2.7415*e* − 1	2.2196*e* + 0	2.4281*e* − 1	2.2226*e* − 1	1.7920*e* + 0	3.3701*e* − 1	8.5198*e* − 1
UF7	10000	7.7783*e* − 1	2.7314*e* − 1	2.2618*e* + 0	2.3713*e* − 1	2.1070*e* − 1	1.8708*e* + 0	3.1525*e* − 1	8.7601*e* − 1
UF8	10000	7.6917*e* − 1	3.9688*e* − 1	6.0293*e* + 0	3.2237*e* − 1	2.1981*e* − 1	1.5660*e* + 0	3.9585*e* − 1	2.0080*e* + 0
UF9	10000	8.0635*e* − 1	4.1594*e* − 1	4.4678*e* + 0	3.2879*e* − 1	2.2249*e* − 1	1.6600*e* + 0	3.9587*e* − 1	1.8525*e* + 0
UF10	10000	7.1560*e* − 1	3.3209*e* − 1	2.6416*e* + 0	2.5976*e* − 1	2.2396*e* − 1	1.6415*e* + 0	3.5417*e* − 1	1.1690*e* + 0

**Table 7:** Runtime of different MOEAs and GCDMOPSO on ZDT1–ZDT4 and ZDT6, DTLZ1–DTLZ5, and UF1–UF10 problems, respectively.

Problems	*FEs*	NSGA-II	NSGA-III	MOEAD	MOEAIGDNS	SPEA2	SPEAR	IBEA	GCDMOPSO
ZDT1	10000	3.4758*e* − 1	5.3468*e* − 1	3.4007*e* + 0	6.4332*e* − 1	8.0079*e* + 0	8.6639*e* + 0	1.0740*e* + 1	4.2437*e* + 0
ZDT2	10000	6.7123*e* − 1	4.8913*e* − 1	3.3432*e* + 0	3.6390*e* − 1	7.9921*e* + 0	8.5811*e* + 0	1.0624*e* + 1	4.7586*e* + 0
ZDT3	10000	2.4592*e* − 1	4.3497*e* − 1	3.3789*e* + 0	6.4406*e* − 1	8.0529*e* + 0	8.6078*e* + 0	1.0731*e* + 1	3.2039*e* + 0
ZDT4	10000	3.5868*e* − 1	6.3845*e* − 1	3.2340*e* + 0	3.6940*e* − 1	8.1632*e* + 0	8.5680*e* + 0	1.0601*e* + 1	4.3560*e* + 0
ZDT6	10000	2.7101*e* − 1	4.3891*e* − 1	3.2598*e* + 0	3.0581*e* − 1	8.0220*e* + 0	8.6345*e* + 0	1.0646*e* + 1	4.3096*e* + 0
DTLZ1	10000	2.9300*e* − 1	5.3163*e* − 1	3.3872*e* + 0	4.5224*e* − 1	8.1141*e* + 0	8.3389*e* + 0	1.0676*e* + 1	2.7750*e* + 0
DTLZ2	10000	3.2915*e* − 1	8.2367*e* − 1	3.3891*e* + 0	4.6041*e* + 1	1.4410*e* + 1	8.9867*e* + 0	1.2031*e* + 1	3.1383*e* + 0
DTLZ3	10000	3.3083*e* − 1	5.3091*e* − 1	3.6684*e* + 0	4.5267*e* − 1	8.6110*e* + 0	9.2516*e* + 0	1.2214*e* + 1	1.5740*e* + 0
DTLZ4	10000	3.6689*e* − 1	6.9115*e* − 1	3.7662*e* + 0	4.0429*e* + 1	1.4007*e* + 1	8.9418*e* + 0	1.1746*e* + 1	0.8514*e* + 0
DTLZ5	10000	3.6991*e* − 1	7.7597*e* − 1	3.7441*e* + 0	1.5471*e* + 1	1.1243*e* + 1	8.9396*e* + 0	1.1829*e* + 1	1.7879*e* + 0
UF1	10000	3.1563*e* − 1	4.9168*e* − 1	3.9203*e* + 0	4.7115*e* − 1	9.1414*e* + 0	9.5004*e* + 0	1.2108*e* + 1	1.0027*e* + 0
UF2	10000	2.8833*e* − 1	4.9032*e* − 1	4.0969*e* + 0	8.5106*e* − 1	9.1164*e* + 0	9.5507*e* + 0	1.2087*e* + 1	1.7896*e* + 0
UF3	10000	3.2962*e* − 1	5.1931*e* − 1	4.0620*e* + 0	5.5841*e* − 1	9.1166*e* + 0	9.6254*e* + 0	1.2138*e* + 1	1.3945*e* + 0
UF4	10000	2.9796*e* − 1	5.5766*e* − 1	4.0546*e* + 0	1.0266*e* + 0	9.0826*e* + 0	9.6639*e* + 0	1.1300*e* + 1	1.7119*e* + 0
UF5	10000	3.0116*e* − 1	5.8167*e* − 1	3.9241*e* + 0	3.7765*e* − 1	8.7468*e* + 0	9.1719*e* + 0	1.2134*e* + 1	7.5826*e* − 1
UF6	10000	3.1538*e* − 1	5.4860*e* − 1	3.8002*e* + 0	4.0976*e* − 1	8.7895*e* + 0	9.3935*e* + 0	1.1596*e* + 1	8.5198*e* − 1
UF7	10000	2.8455*e* − 1	5.3397*e* − 1	3.7370*e* + 0	4.2447*e* − 1	8.6857*e* + 0	9.3448*e* + 0	1.1451*e* + 1	8.7601*e* − 1
UF8	10000	3.5545*e* − 1	6.8885*e* − 1	3.6264*e* + 0	1.8384*e* + 0	8.6025*e* + 0	9.2178*e* + 0	1.1520*e* + 1	2.0080*e* + 0
UF9	10000	3.5060*e* − 1	6.9686*e* − 1	4.2788*e* + 0	1.3978*e* + 0	8.6964*e* + 0	9.0618*e* + 0	1.1917*e* + 1	1.8525*e* + 0
UF10	10000	3.4883*e* − 1	6.7182*e* − 1	4.2034*e* + 0	1.0353*e* + 0	8.7814*e* + 0	9.1103*e* + 0	1.1806*e* + 1	1.1690*e* + 0

## Data Availability

No data were used to support this study.
